# Corpus Luteum

**Published:** 1846-12

**Authors:** 


*Corpus Luteum. Its value as evidence of conception; and its relations to Legal Medicine, with the characteristics of the True and False. Being an attempt to reconcile the conflicting opinions of writers by the recent discoveries in the Physiology of the Ovaries.* By Samuel S. Purple, M. D.

This is the title of a short paper re-published in a pamphlet form, from the November No. of the New-York Journal of Medicine. We have, in the previous numbers of this Journal, adduced the late discoveries respecting the maturation and periodical discharge of ova, and the consequent presence in the ovaries of *corpora lutea* which are not to be regarded as signs of conception. These are called *false corpora lutea,* in distinction from those which are, in reality, to be considered as evidences of conception—the latter being termed the *true corpora lutea.* It is obviously a matter of interest and importance to be prepared to distinguish between the two. We quote Dr. Purple's summary of the characters appertaining to each, respectively.—*Ed. Buff. Jour.*

"1. As to *external form,* being an increase in the size of the ovary when compared with its opposite, sometimes amounting to nearly one half, which seems like the addition of a portion to the substance of the ovary. The prominence of a dark color, varying from a deep red to a dark blue or purple. Upon the summit an excavation, or depression, leading to an opening into a cavity below

(this last is the appearance in the early stages), and running over the surface a number of red toruous lines.

2. As to *size* and *form*. When cut into, varying according to the age from conception, generally from five to eight lines in length, being in form irregular, round or oval, and possessing a cavity or a stellated line or cicatrix.

3. As to *color*, varying also according to the period examined, if previous to the third or forth month, frequently of a reddish brown or of a light yellow—the centre lining the cavity of a dark red. If examined towards the close of gestation, the color approaches a light or greyish yellow, having generally a white, radiated or stelliform line of a pearl color in the place of the cavity. We have noticed in one case at the full period a large cavity filled with a dark fluid, the cavity surrounded by the yellow matter about an eighth of an inch in thickness.

4. As to *structure*—which appears to be glandular, resembling somewhat a section of the human kidney, or that portion of the brain called by anatomists, *centrum ovale*. It is vascular, and capable of receiving a fine injection when thrown into the spermatic arteries.

These may be said to be the leading features or characteristics of a *true corpus luteum*.

Those of the false corpora lutea are:

1. The want of any elevation or enlargement of the ovary.

2. *The size*, which may vary, being from that of a pea down to the size of a millet seed, when it appears like a spot in the substance of the ovary.

3. *The color*, which is often of a dirty, at other times of a light yellow or grey color. It wants the glandular appearance and the central cavity lined by smooth membrane.

4. In the fact of its not being capable of receiving an injection. Dr. Montgomery has given, as one of the characteristics, the absence of the external cicatrix. "The external cicatrix," says he, "is almost always wanting." This is the case in those which arise from an enlarged Graafian vesicle, and by the subsequent absorption of the fluid, producing a puckered or shrivelled appearance; but is not the case in those which arise from a ruptured Graafian vesicle during menstruation, *which is by far the most common form of false corpora lutea*."

With respect to the reliance to be placed on the presence of *true corpora lutea* as evidences of impregnation, Dr. Purple's opinion is thus expressed:

"From the numerous examinations which we have made of the ovaries of human females, at all ages and under all conditions of health and disease, during childhood, at puberty, before, during and after menstruation, at the various periods of gestation, and after delivery, we are firmly of the belief that the *true corpus luteum is*

*an infallible sign of conception;* and that the great diversity of opinions which have been put forth relating to the value of this body as an evidence of conception, has arisen from the want of a proper knowledge of the physiology of the ovaries, consequently confounding and calling all yellow bodies or spots corpora lutea.

From what we have read of the writings of those who differ from this opinion, we can but believe that they have mistaken the true appearance of the corpus luteum, and hence have confounded, in their descriptions, the *false* with the *true*, either forgetting, or not knowing that all yellow bodies are not true corpora lutea, and hence are in no way connected with the process of generation."

